# The importance of promoting positive childbirth experiences for women: a perspective paper

**DOI:** 10.3389/fgwh.2025.1599249

**Published:** 2025-07-17

**Authors:** Sigfridur Inga Karlsdottir, Nicky Leap

**Affiliations:** ^1^ Faculty of Nursing, University of Akureyri, Akureyri, Iceland; ^2^ School of Nursing and Midwifery, University of Technology Sydney, Sydney, NSW, Australia

**Keywords:** childbirth, empowerment, experience, positive, women

## Abstract

Childbirth can be a profound and transformative experience, one that embodies complex emotional challenges. Childbirth experiences can have profound and lasting consequences, both positive and negative, shaping a woman's physical, emotional, and psychological well-being. A positive childbirth experience often fosters feelings of empowerment and birth may carry a sense of accomplishment and strength into motherhood. This can enhance bonding with the baby, reduce the likelihood of postpartum depression, and contribute to an overall positive transition into parenting. Supportive environments, effective pain management, and respectful care from healthcare providers play critical roles in creating such experiences. In contrast, a negative childbirth experience can have significant adverse effects. Negative childbirth experiences caused by, for example, a lack of control, disrespectful treatment, or traumatic events during childbirth may lead to feelings of failure, fear, or even post-traumatic stress disorder. These can undermine maternal self-esteem, strain relationships, and hinder the mother-baby bond. Addressing both women's positive and negative childbirth experiences requires providing compassionate, individualised care, fostering open communication, and ensuring that all women feel heard, valued, and supported throughout their childbirth journey. When planning care for women and their families through the childbirth process, it is crucial for health care providers to understand women's perspectives and know how to maximise the likelihood of a positive childbirth experience. This paper explores the significant impact of a positive childbirth experience on a woman's life. It offers perspectives on the importance of recognising and measuring women's childbirth experiences in the ongoing development of maternity service provision.

## Introduction

Childbirth is a profoundly transformative experience that can encompass a range of emotions, from joy and empowerment to vulnerability and fear. A positive childbirth experience can enhance a woman's confidence, emotional well-being, and physical recovery, fostering a strong bond with her baby. On the other hand, negative experiences, arising, for example, from a lack of control, disrespectful treatment, or traumatic events, can lead to psychological distress, including postpartum depression or post-traumatic stress disorder.

The World Health Organization (WHO) emphasises the importance of fostering a positive childbirth experience for all women, regardless of the type or setting of the birth. Various WHO publications identify that healthcare providers, and midwives in particular, can play a vital role in shaping positive childbirth experiences by delivering supportive, respectful, and personalised care (1–3).

This perspective paper explores the impact of women's childbirth experiences on their lives and highlights the importance of recognising and measuring these experiences in the planning of maternity services. We argue that features of professionalism in midwifery care that are potentially associated with the promotion of empowerment for new mothers (3) should be incorporated into maternity care planning in an effort to ensure that all women feel heard and valued during this significant period of their lives.

## The importance of measuring and fostering positive childbirth experiences in maternity care systems

It is widely acknowledged that childbirth is challenging and demanding for women, with potential implications for both positive and negative experiences, but this has not necessarily been captured in the measures that are chosen to define the outcomes of childbirth. Birth outcomes tend to be measured by a range of physical outcomes in categories related to the type of labour and birth, for example: spontaneous onset or induction of labour, acceleration of labour, vaginal birth, birth assisted with vacuum extraction or forceps, birth by caesarean section as well as neonatal outcomes. Women's experiences are often not included in these routine outcome measures of childbirth.

In 2018, the WHO published new recommendations for labour care, *Intrapartum care for a positive childbirth experience* (1), followed in 2020 by the *WHO Labour Care Guide User's Manual* (2). In both these documents, a positive childbirth experience is identified as an essential outcome that contributes to the health and well-being of the mother and child (3). The importance of fostering a positive childbirth experience is also a major theme in WHO publications highlighting the imperative to provide “respectful maternity care”: healthcare providers should provide care “to all women in a manner that maintains their dignity, privacy and confidentiality, ensures freedom from harm and mistreatment, and enables informed choice and continuous support during labour and childbirth” (1).

The humanisation of childbirth movement has, among other things, emphasised the importance of a positive childbirth experience to promote women's psychological health after childbirth (4). This has included highlighting the essential role of effective communication between maternity care providers and women in labour, using simple and culturally acceptable methods. Other strategies that have been shown to promote emotional, psychological and physical health for women include systems that facilitate a companion of choice throughout labour and childbirth, continuous midwifery support in labour and in specific contexts, midwifery-led continuity of care throughout antenatal, intrapartum, and postnatal care in settings with well-functioning midwifery programmes (5–7).

## Definitions of a positive childbirth experience

Clear definitions are an important starting point when focussing on which factors may contribute to both positive and negative childbirth experiences (8). A positive childbirth experience has been defined as: “ … an experience that refers to a woman's experience of interactions and events directly related to childbirth that made her feel supported, in control, safe, and respected; a positive childbirth can make women feel joy, confident, and/or accomplished and may have short and/or long-term positive impacts on a woman's psychosocial well-being” (8). On the other hand, a traumatic childbirth experience has been identified as: “ … a woman's experience of interactions and/or events directly related to childbirth that caused overwhelming distressing emotions and reactions, leading to short and/or long-term negative impacts on a woman's health and wellbeing” (9). By using these definitions, it becomes possible to identify and validate positive childbirth experiences, as well as guide improvements in practice, education, research, advocacy, and policymaking (8).

## Why promoting positive childbirth experiences for women matters

People tend to focus more on negative childbirth experiences than positive ones, whether in personal stories, audit, or research. Given the number of women who report negative childbirth experiences and the long-term effects such experiences can have on their health and wellbeing, it is crucial to promote positive childbirth experiences. The prevalence of negative childbirth experiences varies, but studies have suggested that between 6%–44% of women experience childbirth as traumatic (10). A negative experience of childbirth can have a profound effect on a woman's life (11, 12): for example, the identified increase in the likelihood of postnatal depression, stress and anxiety (13, 14) can directly affect postnatal mother-child attachment and negatively influence children's cognitive development (15, 16).

A positive experience of childbirth can also have long-term implications for women's health and well-being, as identified in a qualitative study conducted in Sweden (17). Women who had a very positive birth experience described an increase in their feelings of confidence, ability and strength. A trustful and supportive relationship with their midwife was crucial in promoting this, particularly where the midwife worked as an active guide through pregnancy and labour, helping the woman with physical and mental preparation for birth and techniques to manage labour. A supportive environment for labour, including teamwork between parents and all staff, plus a sense of trust and support from the father of the child, were also important factors in enabling a sense of control and safety. Other studies have also highlighted women's similar views that feeling supported, in control, safe, respected and involved in decision-making during pregnancy, labour and birth were important factors that contributed to their positive birth experiences, including a sense of increased strength and the capacity to cope with new motherhood and other life challenges (6, 7, 18, 19). Women have described their self-esteem increasing as a result of coping with the challenges of labour and birth and that this increased their feeling that they could cope with life overall and an increased responsibility to their family. Women have described becoming more confident in the role following a positive birth experience and are also more likely to choose vaginal birth in subsequent births (20). In contrast, according to Swift et al. (21), women's low birth satisfaction was independently associated with higher symptoms of childbirth-related post-traumatic stress disorder (CB-PTSD); this emphasises the potential effect that a negative birth experience can have on women's lives and how vital a positive childbirth experience is for women's health after childbirth.

For many years, studies have explored the powerful impact a positive birth experience can have on women's lives for years after childbirth in terms of empowerment (17, 22, 23). Empowerment is a complex and multidimensional concept defined by Lafrance and Mailbot (24) as a process of action and reflection that builds parents' confidence, knowledge, and capacity for decision-making. Two processes have been described related to childbirth empowerment: the strengthening of parental skills, and appropriation, which enhances parents' sense of control (20). Positive childbirth experiences can boost women's self-esteem and confidence in their parental role (17) and strengthen women's attachment to their children (15).

## Factors that contribute to positive experiences of childbirth

Women have highly different needs during childbirth as identified in a systematic review of over 40 qualitative studies of women's and their partner's experiences of childbirth in different settings in low, middle and high-income countries (25). Whilst bringing to light the complexity and multidimensionality of the birth experience, the review suggested that what women experience as positive and meaningful for them is surprisingly similar. Parents want support in preparing for birth and facing the challenges of uncertainty around pregnancy, labour and birth; they want sensitive and supportive care during labour. Positive experiences of childbirth are directly related to feeling these needs are met (25), suggesting that support from caregivers and birth attendants is arguably the most crucial ingredient women need during labour and birth (7). According to a Cochrane systematic review, continuous support during labour can lead to several positive outcomes, including: increased spontaneous vaginal birth, shorter duration of labour, and decreased caesarean birth, instrumental vaginal birth, use of any analgesia, use of regional analgesia, low Apgar score and negative feelings about childbirth experiences (6).

Women's childbirth experiences have been studied from many different perspectives, focusing on different outcomes. Positive experiences have been associated with satisfaction with antenatal care and choice of maternity service or provider (22, 26); factors related to personal ability and women's psychological experience of childbirth (27); the predictors of positive childbirth experiences, including the various actors; and positive experiences of managing pain in labour (28).

Recent studies have argued that neurobiological processes induced by the release of oxytocin may contribute to positive experiences of birth and promote an optimal transition to motherhood. The case is made for midwifery one-to-one support in labour and maternity care that optimises the function of neuroendocrine processes, even when birth interventions are used; the importance of providing physical, emotional and social support for women through the intense and transformative psychological experience of birth has the potential to be transformative, particularly where women are encouraged to believe in their ability to give birth and where physiology is not disturbed (29).

The importance of women's experiences fulfilling or exceeding their prior personal and social-cultural beliefs and expectations of childbirth has been identified (30). In a narrative literature review of 20 studies of a positive birth experience of first-time mothers, five themes emerged: women's experiences of personal strength and their ability to give birth, including a sense of pride in how they managed pain and challenges during labour; the support and guidance they received from midwives and obstetricians; physical and mental preparation for the birth; feelings of trust and support from their midwives and partners; involvement in decision-making; and prayers and spiritual support from family and healthcare providers (31).

## Maternity service development to measure and optimise positive experiences for women

There is an imperative to recognise that women's experiences of childbirth are a crucial aspect of measuring and informing high-quality care, offering insight into what truly matters to them, and enabling service planning that aligns with their needs (3, 15). This involvement of women in service development should also be incorporated into midwifery education and staff training (32).

In many countries, women's experiences are not measured or studied. Indeed, in many low-income countries, the maternal mortality rate is almost the only measure available (2). Several different questionnaires have been developed to measure women's childbirth experience. For example, in Sweden (33) the childbirth experience questionnaire consists of four domains: own capacity, professional support, perceived safety and participation. This questionnaire has been translated, tested and validated, for example, in Iceland (5) and the UK (34). The widely used Birth Satisfaction Scale Indicator (BSS-RI) has also recently been revised (BSS-RI) (35).

Midwifery models of care and theories are essential when planning services for childbearing women, as many of them focus on the midwife–woman relationship, woman-centeredness, and a salutogenic approach, factors that women have identified as important in the kind of care they need from midwives (32). The PRIME theory of professionalism in midwifery draws on women's perspectives regarding the competencies and characteristics a midwife needs upon graduation to promote a sense of safety and well-being for women. It defines essential qualities and skills needed to support women and their partners effectively and foster a sense of safety, comfort, and confidence (32). The definition of a positive childbirth experience by Leinweber et al. (8) emphasises the critical role of healthcare providers in facilitating a positive childbirth experience by seeing each woman as unique and responding to her individual needs (25, 26, 30, 36). Where women are treated with respect, and where they have formed trusting relationships with caregivers who have facilitated a relaxing and welcoming atmosphere, they are more likely to have a positive experience of childbirth (31).

During pregnancy, birth and post-partum, it is essential that healthcare providers know how they can strengthen women's self-esteem to deal with childbirth in every possible way to increase the likelihood of a positive childbirth experience and the potential for empowerment. Measuring empowerment is not simple, and in a scoping review of 23 studies including 13 instruments to measure empowerment during pregnancy, the overall findings were that focusing on facilitating women's choice and decision making, women's belief in their abilities and control over situation, self and others is of importance. The instruments included five main components: facilitation of women's choice and decisions, women's belief in their abilities, control over situations, self and others, gender equality and access and control of resources. Under each component are many other components, such as decision-making, self-determination, support and assurance from others, financial authority, self-efficacy, autonomy, and legal dimensions (37).

Some scales are available to measure empowerment, such as the pregnancy-related empowerment scale (PRES) (38) and an empowerment questionnaire (EQ) which focuses on empowerment among pregnant and postpartum women (39). PRES scale has four dimensions: provider connectedness (relationship that minimised the power differential and created with respect and trust), with six statements, skilful decision making (process by which women come to evaluate and choose a direction that will impact their health), with three statements, peer connectedness (a bond between women that develops from the evolution of caring and supportive relationship) with two statements and gaining voice (to be knowledgeable about their health and advocate for their health care options for self and family) with five statements. That tool is a valid and reliable measure of women's health-related empowerment during pregnancy (38). In a scoping review measuring women's empowerment during the perinatal period in high-income countries (40) 21 instruments were identified, and 11 were validated among women during the perinatal period. However, no instrument has been specifically designed for women during the perinatal period that encompasses all dimensions of empowerment and its defining attributes.

## Discussion

It is well established that childbirth experiences can have a significant positive or negative impact on both women and their families. Strategies for enhancing a positive childbirth experience are important in efforts to promote a sense of control, self-efficacy and self-esteem for women (41).

We argue that measuring women's childbirth experiences is essential and should be recognised as a fundamental part of childbirth outcomes. In many high-income countries with well-developed healthcare systems that adhere to international standards and evidence-based practice guidelines, there is no routine measurement of women's childbirth experiences. This underscores a deficiency in data concerning how women perceive childbirth and its effects on their own lives as well as those of their family's following childbirth. In recent years, validated questionnaires have been developed to measure women's childbirth experiences, although many place greater emphasis on negative aspects rather than positive ones. While both perspectives are important, shifting the focus beyond negative experiences can assist healthcare providers in understanding the factors that contribute to a positive birth experience and integrate elements that foster positivity in their daily care of women and their families during childbirth.

Midwives have the unique privilege of supporting families during one of life's most transformative moments when bringing new life into the world. This responsibility must be approached with care, as the quality of services can significantly affect a mother and her family's well-being for years to come. We suggest that the PRIME theory can also be used in the educating and training of midwives so that women's views on what they think are the most important characteristics of a midwife can contribute to optimising the potential for positive childbirth experiences Every midwife and midwifery student should recognise the enduring effects of their care and strive to provide the best possible service to maximise the likelihood of a positive childbirth experience. This aligns with the International Code of Ethics for Midwives, which emphasises human rights, justice, and equal access to care, along with mutual relationships of respect and dignity (42). In their position statement, *Heritage and Culture in Childbearing* (43) the International Confederation of Midwives urges their member associations to work with midwives, women, policy makers and the community to promote positive birth experiences by implementing culturally safe health services. For individual midwives across the world, working in ways that promote cultural safety involves an ongoing process of learning to be open to other people's cultural ways of being, knowing, and doing, whilst acknowledging power dynamics and being open to challenging their own individual culture, attitudes and beliefs (44).

The WHO emphasises respectful maternity care, which refers to care organised and provided for all women that upholds their dignity, privacy, and confidentiality, ensuring freedom from harm and mistreatment while allowing informed choice and continuous support during labour and childbirth. Routinely measuring women's experiences of childbirth and empowerment is crucial and should be valued as an important outcome of childbirth. There is an imperative to continue to develop studies that enable women's voices to contribute to education and maternity service development; this should include qualitative research that enables in-depth exploration of individual women's experiences (45).

## Conclusion

When planning maternity care for women and their families, there is an imperative for health care providers to understand women's perspectives and know about factors that maximise the likelihood of a positive childbirth experience. All maternity care systems should consistently utilise validated questionnaires and research methods to engage in discussions with women about their childbirth experiences. Women's perspectives should inform the development and analysis of outcome measures as well as educational activities, with an aim to provide high quality care and promote positive birth experiences for women and their families. Improved health outcomes and wellbeing can include the strengthening of parental skills and attachment and for women, a sense of empowerment related to self-esteem and increased confidence.

## Data Availability

The original contributions presented in the study are included in the article/Supplementary Material, further inquiries can be directed to the corresponding author.
